# Comparison of failure loads and compressive stress in Press on metal and Press on Y-TZP copings

**DOI:** 10.12669/pjms.36.7.2472

**Published:** 2020

**Authors:** Fahim Vohra, Mohammed Bin Shuwaish, Modhi Al Deeb, Rana Alhamdan, Naif Alotaibi, Tariq Abduljabbar

**Affiliations:** 1 Fahim Vohra, Department of Prosthetic Dental Science, College of Dentistry, King Saud University, Riyadh 11545, Saudi Arabia; 2 Mohammed Bin Shuwaish, Department of Restorative Dental Science, College of Dentistry, King Saud University, Riyadh 11545, Saudi Arabia; 3 Modhi Al Deeb, Department of Prosthetic Dental Science, College of Dentistry, King Saud University, Riyadh 11545, Saudi Arabia; 4 Rana Al Hamdan, Department of Restorative Dental Science, College of Dentistry, King Saud University, Riyadh 11545, Saudi Arabia; 5 Naif Alotaibi, Department of General Dentistry, College of Dentistry, King Saud University, Riyadh 11545, Saudi Arabia; 6 Tariq Abduljabbar, Department of Prosthetic Dental Science, College of Dentistry, King Saud University, Riyadh 11545, Saudi Arabia

**Keywords:** Failure load, Compressive stress, Metal copings, Hot pressing, Y-TZP, Crowns

## Abstract

**Objective:**

The aim of the study was to assess the failure loads and compressive stresses among bilayered press on Y-TZP (POZ) and press on metal (POM) crowns with different core-veneer thickness.

**Methods:**

Thirty metal and Y-TZP copings were fabricated using CAD-CAM technology with specified thickness. All copings were veneered with ceramic materials using hot pressing technique, with 2mm and 2.5mm thickness. The different coping veneer thickness of crowns resulted in six study groups, including, POM: Coping/ veneer thickness of 0.7/2mm (Gp1), 0.7/2.5mm (Gp 2) and 1mm/2mm (Gp 3)-POZ: 0.7/2mm (Gp A), 0.7/2.5mm (Gp B) and 1mm/2mm (Gp C). Crowns were cemented to a standard implant analog and failure loads (FL) and compressive stress (CS) was ascertained by controlled load application in a universal testing machine. Data was analysed using ANOVA and multiple comparisons test.

**Results:**

The maximum FL were observed in the POM specimens with a C/V ratio of 1/2 (Group 3-1880.67± 256.78 N), however the lowest FL were exhibited by POZ crowns with 1/2 C/V ratio (Group C-611.89± 72.79 N). Mean FL and CS were significantly higher in POM compared to POZ crowns in respective groups. Increasing the coping-veneer thickness increased FL and CS among POM crowns. Increasing veneer and decreasing coping thickness improved FL and CS among POZ crowns.

**Conclusions:**

Press on metal specimen showed higher resistance to fracture than Press on Y-TZP specimens. Improved failure loads were observed in thin coping and thick veneers among Press on Y-TZP crowns.

## INTRODUCTION

All ceramic restorations are commonly used in esthetic dentistry. They offer excellent translucency and biocompatibility for long-term oral rehabilitations. Yttrium stabilized tetragonal zirconia polycrystal (Y-TZP) materials based crowns and fixed partial dentures, combine the mechanical strength of the coping and the esthetic appearance of translucent ceramic veneer. However the fracture and chipping of veneering ceramic, compromises the prognosis of Y-TZP prosthesis as reported in previous studies.^1^,^2^ The failures occurring in the veneer or at the core-veneer interface is attributed to the presence of tensile stresses trapped within the veneer.^3^,^4^ The stresses are higher in the Y-TZP specimnes compared to metal ceramic prosthesis, resulting in higher failures in bilayered Y-TZP.^3^-^5^ Mutiple factors are reported to influence the weakening internal stresses including, coping surface treatments, coefficient of thermal expansion compatibility, veneer coping thickness, veneer application technique and heat treatments.^6^-^10^

The conventionally used layering technique for veneering Y-TZP using inherently weaker glass ceramics potentially induce surface flaws and shrinkage. Consequently, micro cracks and porosities originating from the inner and outer surface of bilayered restorations result in damage and failure. Hot pressing is another technique for veneering zirconia-based Y-TZP ceramics. As it involves the use of pre-sintered ceramic ingots and lost wax technique, it avoids multiple firings, minimizing the internal flaws of ceramic and shrinkage also allowing greater control on anatomical characterization and fit.^11^ Earlier studies have shown similar fracture toughness for layered and pressed veneers for Y-TZP restorations, however more consistency was observed in hot pressed crowns.^3^,^4^ In addition, thickness of materials (coping and veneer), influences the fracture toughness of Y-TZP bilayered ceramics. Reports have recommended the use of metal ceramic crowns (MCC) design to be implemented in fabrication of Y-TZP copings (0.5-0.7mm coping and 1.5-2.0 mm ceramic.^6^ However increased tensile stresses have been demonstrated in ceramic copings that may not adequately support the overlying porcelain.^5^ Increased thickness of the ceramic coping on the other hand has shown a compromising influence on the resulting stresses and toughness of veneering ceramics in a bilayered Y-TZP complex.^3^,^4^,^11^

A contemporary technique is pressing of ceramic veneers on the metal copings (Press on metal-POM) to combine the benefits of better restorative contours and strength of metal copings. However, the influence of different veneer-coping thickness and veneering technique in comparison to bilayered Y-TZP specimens, for POM specimens is not known. It is hypothesized that POM crowns will show higher failure loads and compressive stress than press on zirconia (POZ) crowns. In addition, in comparison to POZ crowns, coping and veneer thickness will show mechanical improvements in POM crowns. Therefore, the aim of the study was to assess the failure loads and compressive stresses among bilayered press on Y-TZP and press on metal crowns with different core-veneer thickness.

## METHODS

The project was performed in accordance with the checklist of reporting in vitro studies (CRIS) guidelines.

### Fabrication of copings

An implant abutment analog (Nobel Biocare, Kloten Switzerland) with dimensions of 5.5mm height, 6° taper and 1.5mm collar height was mounted in acrylic resin vertically. Abutment was scanned using Cercon Eye scanner (DeguDent GmbH, 63457 Hanau-Wolfgang, Germany) and using Cercon Brain milling machine and Cercon Base blanks (Y-TZP) material, thirty Y-TZP copings were fabricated. Twenty copings had 0.7mm and ten had 1mm occlusal thickness. The copings had a cement gap of 0.05mm and 1mm thick axial walls. The milled copings are placed in a Heat furnace (Cercon Heat, DeguDent GmbH, 63457 Hanau-Wolfgang, Germany) and sintered (170 minutes - 1600 °C).

The metal copings were designed using Cercon Art software according to the STL file from the previous scan. Using a milling machine (Ceramill Motion 2- DeguDent GmbH, Hanau-Wolfgang, Germany) and Ceramill Sintron alloy blanks (Co-Cr- Amann Girrbach AG Herrschaftswiese, Koblach, Austria) twenty 0.7mm and ten 1mm metal copings were fabricated with the same dimensions as Y-TZP copings.

### Ceramic Veneer application

All copings were veneered using hot pressing method. Among Y-TZP copings, wax-ups of 2mm was performed on ten 0.7mm and ten 1mm copings however, a wax build-up of 2mm was performed on ten 1mm copings. Similarly, among metal alloy copings, wax-ups with similar dimensions were performed. All wax-ups were overbuilt by 0.2mm for later finishing of ceramic surfaces. This resulted in six study groups of 10 specimens each.

Group 1: POM (Press on metal). Coping/ veneer thickness of 0.7/2mm

Group 2: POM (Press on metal). Coping/ veneer thickness of 0.7/2.5mm

Group 3: POM (Press on metal). Coping/ veneer thickness of 1/2mm

Group A: POZ (Press on zirconia). Coping/ veneer thickness of 0.7/2mm

Group B: POZ (Press on zirconia). Coping/ veneer thickness of 0.7/2.5mm

Group C: POZ (Press on zirconia). Coping/ veneer thickness of 1/2mm

An IPS e.max Ceram ZirLiner (Ivoclar Vivadent, AG, Schaan / Liechtenstein) was applied to all Y-TZP copings and fired, prior to wax up. Following wax up and wax burn out, veneering ceramic (IPS e.max ZirPress, Ivoclar Vivadent, AG, Schaan / Liechtenstein) pre-sintered ingots were hot pressed on the Zr copings to the desired dimensions. All metal copings were air-abraded prior to wax-up and veneering ceramic (IPS Inline POM, Ivoclar Vivadent, AG, Schaan / Liechtenstein) pre-sintered ingots were pressed on the metal alloy copings to the desired dimensions. The processing temperatures for Zirpress and POM materials were 700°C to 935 °C and 835°C to 1000°C respectively. All coping surfaces were flattened and finished to required dimensions using abrasive and ceramic polishing discs on a surveyor. All specimens were exposed to thermocycling (5°C and 55°C water baths for 10000 cycles- 25sec to 5sec) and cemented to abutment analog with zinc oxide eugenol (Temp Bond NE- Kerr, 6934 Bioggio, Switzerland) under a static load of one kg for three minutes. A single experienced technician fabricated all samples.

### Failure load and compressive stress assessment

Cemented specimen were mounted on the universal testing machine (Instron- 5965) and secured with a metal clamp. A round-ended probe was vertically placed on the occlusal surfaces of specimens and controlled load was applied at a crosshead speed of 0.5mm per minute until fracture. The compressive stress at fracture and maximum failure loads were recorded in Megapascals (MPa) and Newton respectively. The mode of fracture within veneer ceramic or delamination was also tabulated. FV and NO performed the fracture testing mechanically.

### Statistical Analysis

A statistical software (SPSS- version 20) was used to tabulate data and normality assessment was performed by Kolmogorov-Smirnov test. ANOVA was performed to compare overall means, and multiple comparisons test (post-hoc Tukey) was used to compare individual group means of outcomes.

## RESULTS

**Fig.1:**
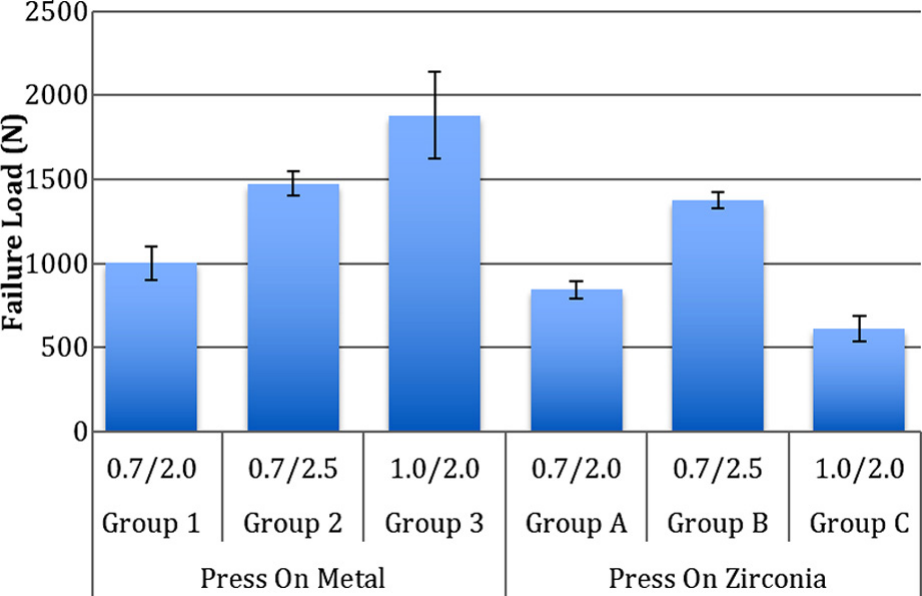
Comparison of Failure loads among study groups.

**Fig.2:**
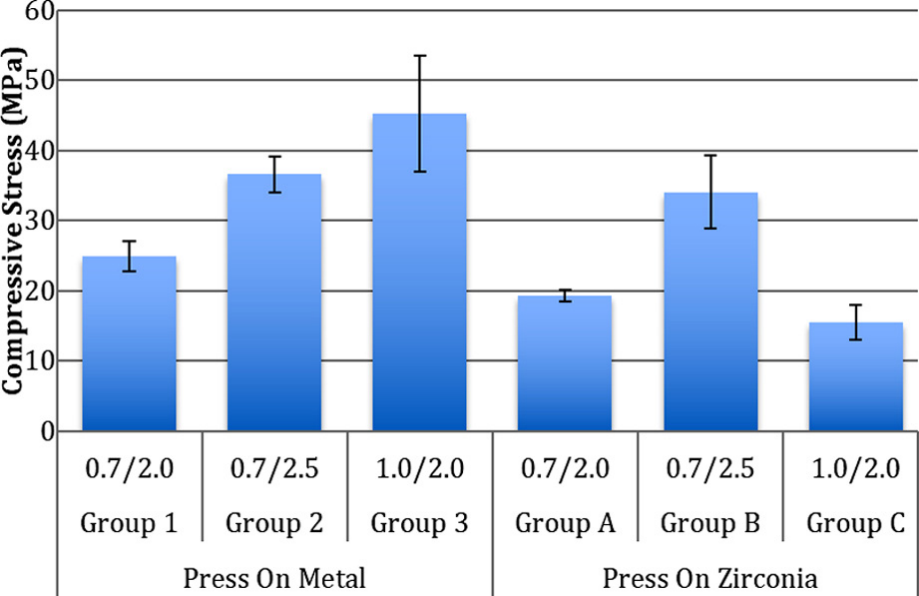
Comparison of compressive stress among study groups.

The data obtained from mechanical testing for the included study groups was normally distributed. The maximum failure loads were observed in the POM specimens with a C/V ratio of 1/2 (Group 3-1880.67± 256.78 N), however the lowest failure loads were exhibited by POZ (Press on zirconia) crowns with 1/2 C/V ratio (Group C-611.89± 72.79 N) (Table-I). Mean failure loads among POM crowns ranged from 1000.82 ± 101.59 N to 1880.67 ± 256.78 N. There was significant difference among the failure loads in POM samples with different C/V ratios (p<0.05). Increasing the coping thickness from 0.7 mm (Group 1) to 1mm (Group 3) increased the failure loads significantly (p<0.05) (Fig.1). Increasing the veneer thickness from 2mm (Group 1) to 2.5mm (Group 2) increased the failure loads significantly (p<0.05), however the influence was less than the effect of increasing the coping thickness (Group 3). Compressive stresses at failure among the POM specimens were highest in group 3 (45.22± 8.22 MPa) and the lowest in group 1(24.91± 2.08 MPa) (Table-II). Compressive stress increased as the veneer thickness (group 2- C/V-0.7/2.5) and coping thickness (group 3- C/V-1/2) increased, as compared to control (group 1-C/V- 0.7/2) (Fig.2). Compressive stresses were significantly different among the POM crowns with different C/V ratios (p<0.05) (Table-II).

**Table-I:** Maximum failure loads among the crowns included in the study groups.

Study Groups	Press On Metal			Press On Zirconia			ANOVA*

	Group 1 0.7/2.0	Group 2 0.7/2.5	Group 3 1/2	Group A 0.7/2.0	Group B 0.7/2.5	Group C 1/2		
Mean	1000.82^a^	1473.47^b^	1880.67^c^	843.43^d^	1376.31^e^	611.89^f^	p<0.01
SD	101.59	73.20	256.78	51.62	50.21	72.79	

Dissimilar alphabets denote significant difference among groups (Tukey post hoc test)

**Table-II:** Maximum compressive stress among the crowns included in the study groups.

Study Groups	Press On Metal			Press On Zirconia			ANOVA*

	Group 1 0.7/2.0	Group 2 0.7/2.5	Group 3 1/2	Group A 0.7/2.0	Group B 0.7/2.5	Group C 1/2		
Mean	24.91^a^	36.60^b^	45.22^c^	19.34^d^	34.05^b^	15.5^e^	p<0.01
SD	2.08	2.52	8.22	0.81	5.23	2.4	

Dissimilar alphabets denote significant difference among groups (Tukey post hoc test)

Maximum and minimum failure loads among POZ crowns were exhibited by group B (C/V- 0.7/2.5-1376.31± 50.21 N) and group C (C/V-0.7/2- 611.89± 72.79 N) specimens respectively (Table-I). Failure loads among POZ crowns were significantly different (p<0.05) (Fig.1). Increasing the veneer thickness from 2mm (group A- 843.43 ± 51.62 N) to 2.5mm (group B-1376.31 ± 50.21 N) increased the failure loads significantly (p<0.05). Increasing the coping thickness from 0.7mm (Group A- 843.43 ± 51.62 N) to 1 mm (Group C-611.89 ± 72.79 N), significantly reduced the failure loads among specimens (p<0.05). Compressive stress among POZ specimens was significantly higher in group B (34.05± 5.23 MPa) as compared to group A (19.34± 0.81 MPa) and group C (15.5± 2.4 MPa) specimens (p<0.05) (Table-II). Compressive stresses in POZ crowns differed significantly (ANOVA).

Failure loads among the POM specimens were significantly higher than POZ specimens in the corresponding C/V ratio groups (p<0.05) (Fig.2). Compressive stresses at failure among POM specimens were significantly higher than POZ specimens for C/V ratios of 0.7/2.0 (Groups 1 and A) and 1/2 (Groups 3 and C) (p<0.05) respectively. Compressive stresses between POM and POZ specimens of group 2 and Group B were comparable (p>0.05).

## DISCUSSION

The present study was based on the hypothesis that POM crowns will show higher failure loads and compressive stress than POZ crowns. In addition, in comparison to POZ crowns, coping and veneer thickness will show mechanical improvements in POM crowns. The first hypothesis was accepted as POM showed higher outcomes (failure loads) than POZ specimens. The second hypothesis was also accepted as increasing veneer and coping thickness increased failure loads for POM crowns. However, POZ crowns showed a reduction in failure loads with increase in coping thickness. Multiple explanations can explain these findings, including the formation of residual stresses, the crystalline structure and behaviors of ageing of Y-TZP, CTE mismatch between coping and veneer materials and failure dynamics.

Coefficient of thermal expansion (CTE) compatibility is critical for the improved compressive stresses within the core-veneer complex of bilayered restorations.^12^-^14^ The materials (IPS Emax-Zirpress & IPS Press-on-metal) used for core and veneer among POM and POZ materials were compatible. A hot pressing technique was employed for veneering the copings in both metal and Y-TZP materials, to minimize the influence of multiple firing cycles and internal and external flaws, critical for developing stresses within the complex.^3^ Failure loads and compressive stresses were assessed in the specimens as it is representative of the occlusal loads and masticatory function. Coping to veneer thickness of 0.7 mm and 2.5 mm were assessed in the present study as they are used frequently for implant restorations in cases of moderate to severe bone loss.^15^,^16^

In the present study, the failure loads and compressive stress among POM samples were significantly higher than POZ samples in respective coping-veneer groups. Previous studies have suggested higher fracture toughness of metal ceramic compared to Zr ceramic crowns.^3^,^4^ In addition, the veneering material for POM samples are leucite crystal based ceramic (IPS Inline POM).^17^ Leucite crystals act as crack stoppers and prevent crack propagation which improves the fracture resistance of the ceramic material.^18^ By contrast, the veneer ceramic material for POZ crowns was flouro-apatitie based glass ceramic (IPS Zirpress). This material is primarily used with Y-TZP copings due to CTE compatibility. However, the smaller crystal size and higher internal tensile stresses within the veneer among the POZ crowns are possible explanations for its lower failure loads.^19^

Residual stresses within the coping-veneer Y-TZP complex have been reported previously.^20^,^21^ These stresses are locked in within the coping veneer complex without external application of loads and increase the susceptibility of ceramics to crack propagation and lower resistance to fracture.^22^,^23^ By contrast presence of compressive stress improves resistance to fracture. In addition, as the veneer thickness increases with a constant coping thickness, the compressive stresses are enhanced as reported by Mainjot et al,^20^ therefore potentially improving the resistance to fracture. Interestingly in the present study, increasing the veneer thickness for both POZ and POM specimens significantly improved the failure loads and compressive stresses, therefore validating the previous hypothesis with physical data.

Another interesting aspect of Y-TZP in contrast to metal alloys is the low thermal conductivity and transfer of heat from the coping to the veneering ceramic during hot-pressing. In the present study increasing coping thickness improved failure loads for POM specimen, however reduced failure loads in POZ crowns. A possible explanation is the formation of a horizontal thermal gradient at the center of the veneer during solidification of ceramic in thicker Y-TZP specimens.^3^,^4^ The improvement in the failure loads and stress among POM specimens is attributed to the thermal CTE mismatch between the coping and veneer materials, hence reducing crack lengths and improving resistance to failure.^24^,^25^

The outcomes of the study suggest that POM technique for veneering metal ceramic crowns result in higher failure loads than POZ crowns. In addition, increasing the coping veneer thickness improved failure loads for POM crowns. These outcomes suggest increased clinical potential for POM as compared to POZ crowns. However, it is pertinent to mention that the applied forces in this in-vitro experiment were static, axial and dry in contrast to the oral conditions. In addition, the occlusal surface of the crowns exposed to failure loads were without contours (cusp and fossa), due to the limitations of the in-vitro conditions. Therefore, further randomized controlled trials investigating the influence of veneer and coping thickness in POM and POZ crowns are recommended.

## CONCLUSIONS

Failure loads and compressive stresses were significantly higher in press on metal compared to press on zirconia crowns. Increasing veneer and coping thickness improved the failure loads in POM crowns. A thin coping and thick veneer provided maximum failure loads and compressive stress among POZ crowns.

### Authors’ Contribution:

**FV:** Data collection, study design, manuscript writing, final manuscript approval, accountable for work.

**TA:** Data collection, study design, manuscript drafting, data analysis, manuscript approval, accountable for work.

**MBS & NA:** Specimen design and preparation, Data collection, manuscript approval and data interpretation.

**MA & RA:** Data collection, writing, revise, editing, final manuscript approval, table and figure designing
